# Role of the DDX11 DNA Helicase in Warsaw Breakage Syndrome Etiology

**DOI:** 10.3390/ijms22052308

**Published:** 2021-02-25

**Authors:** Diana Santos, Mohammad Mahtab, Ana Boavida, Francesca M. Pisani

**Affiliations:** 1 Istituto di Biochimica e Biologia Cellulare, Consiglio Nazionale delle Ricerche, Via P. Castellino 111, 80131 Naples, Italy; diana.santos@ibbc.cnr.it (D.S.); md.mahtab@ibbc.cnr.it (M.M.); ana.boavida@ibbc.cnr.it (A.B.); 2 Dipartimento di Scienze e Tecnologie Ambientali, Biologiche e Farmaceutiche, Università degli Studi della Campania Luigi Vanvitelli, Via Vivaldi 43, 81100 Caserta, Italy

**Keywords:** DDX11, DNA helicase, DNA replication, G-quadruplexes, sister chromatid cohesion, cohesinopathies

## Abstract

Warsaw breakage syndrome (WABS) is a genetic disorder characterized by sister chromatid cohesion defects, growth retardation, microcephaly, hearing loss and other variable clinical manifestations. WABS is due to biallelic mutations of the gene coding for the super-family 2 DNA helicase DDX11/ChlR1, orthologous to the yeast chromosome loss protein 1 (Chl1). WABS is classified in the group of “cohesinopathies”, rare hereditary diseases that are caused by mutations in genes coding for subunits of the cohesin complex or protein factors having regulatory roles in the sister chromatid cohesion process. In fact, among the cohesion regulators, an important player is DDX11, which is believed to be important for the functional coupling of DNA synthesis and cohesion establishment at the replication forks. Here, we will review what is known about the molecular and cellular functions of human DDX11 and its role in WABS etiopathogenesis, even in light of recent findings on the role of cohesin and its regulator network in promoting chromatin loop formation and regulating chromatin spatial organization.

## 1. Introduction

**Figure 1:**
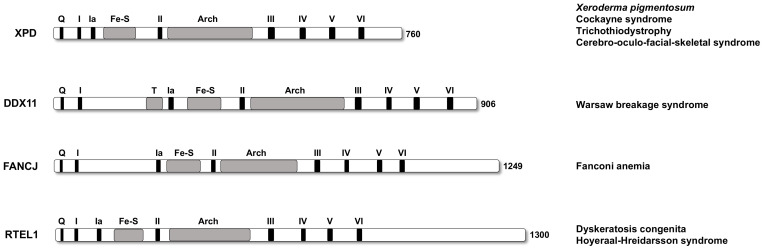
Human super-family 2 DNA helicases containing a Fe-S cluster. A schematic representation of the DNA helicase polypeptide chain is reported. Color code is as follows: *black*, conserved helicase motifs (Q motif, *Q*, and helicase boxes from I to VI); *grey*, Timeless-binding motif (*T*) [9], Fe-S cluster (*Fe-S*) and Arch domain (*Arch*) domain. The related rare hereditary diseases are indicated for each DNA helicase.

*CHL1*/*CTF1*, the gene coding for the yeast counterpart of human DDX11, was identified in a genetic screen of *Saccharomyces cerevisiae* mutants displaying reduced chromosome transmission fidelity (*CTF*) and a consequent chromosome loss (*CHL*) phenotype. The corresponding protein, named Chl1 (or Ctf1), was found to contain all the helicase sequence boxes (from I to VI) that are characteristic of super-family 2 (SF2) DNA helicases [1,2]. The presence of an iron-sulfur (Fe-S) cluster is characteristic of three additional human SF2 DNA helicases: *Xeroderma pigmentosum* group D (XPD) protein, FANCJ and RTEL1. All these DNA helicases are implicated in genome stability maintenance pathways and are genetically linked to rare hereditary diseases and cancer predisposition (see Figure 1) [3]. DDX11/ChlR1, the human ortholog of budding yeast Chl1 protein, was produced in the recombinant form either in mammalian or in baculovirus-infected insect cells and demonstrated to possess an ATPase-dependent DNA helicase activity with a 5′ to 3′ directionality and specific substrate and reaction condition requirements [4,5,6,7].

Herein, we describe the enzymatic properties of human DDX11 DNA helicase, its functions in DNA replication and DNA repair pathways, chromatin structure/dynamics and sister chromatid cohesion. Besides, hypotheses formulated about the etiopathogenesis of Warsaw breakage syndrome (WABS), the hereditary syndrome due to biallelic mutations of the *DDX11* gene [8], are discussed in light of recent findings of its multi-faceted cellular role.

## 2. Localization of DDX11 during Cell Cycle

The localization of DDX11 in mammalian cells in different stages of the cell cycle was for the first time analyzed by the Androphy’s group in 2006 by indirect immunofluorescence experiments with specific antibodies [10]. This work revealed that DDX11 has a sparse nuclear localization in interphase cells from an asynchronous population. On the other hand, in synchronized diploid primary immortalized retinal epithelial cells (RPE1) DDX11 localization appeared to be dynamic: during the early stage of the M phase the protein was found at the condensed chromatin; during metaphase it associated with the spindle poles and fibers; at later stages (until telophase) it localized at the midbody and then, after cell division, it returned to the nuclear compartment. These results were corroborated by co-staining experiments where an anti-DDX11 specific antibody was used in combination with an anti-γ tubulin or an anti-Aurora B antibody, as specific markers of the mitotic spindle and the midbody, respectively, and were confirmed in other mammalian cell types, including HeLa, Cos-7 and C33a [10]. Moreover, in the same study it was also observed that depletion of DDX11 by a tetracycline-inducible RNA interference system caused a prometaphase arrest with a significant percentage of cells with chromosomes dispersed over the entire spindle, as found in early stages of metaphase. Time-lapse imaging of DDX11-depleted cells, where DNA was labeled with Syto13, revealed that a stable metaphase configuration was not reached and after approximately 3 h of the prometaphase block, the chromatin eventually decondensed without chromosomal segregation to the opposite mitotic spindle poles [10]. Of note, during the early stage of mitosis the cohesin subunits Smc1 and SA1 were found at spindle poles where they are recruited via interaction with NuMa, a protein required for mitotic spindle organization; and their depletion in HeLa cell extracts inhibited mitotic aster assembly [11]. These results indicate that DDX11 (together with the cohesin complex) could have a role in mitotic spindle aster assembly and cell division, even if anomalies in spindle morphology or defects in cytokinesis were not observed in DDX11-depleted cells.

More recently, a study carried out by Sun and colleagues showed that DDX11 is a nucleolar protein, as revealed by immunofluorescence experiments using both a mouse monoclonal and a rabbit polyclonal antibody in HeLa and HEK 293T cells [12]. These authors reported that endogenous DDX11 colocalized with nucleolin, an established nucleolus marker, in nuclei of these cell lines. In addition, Western blot analysis revealed the presence of DDX11 also in the nucleolar fraction of HeLa cell extracts. However, in very recent studies it was found that a great proportion (if not the majority) of the DDX11 protein is present in the cytoplasmic and not in the nuclear fraction of human cell extracts [13,14]. A possible localization of DDX11 at a specific cytoplasmic organelle/compartment needs to be more carefully analyzed and might lead to the discovery of novel cellular functions of this protein.

## 3. Two Human *CHL1* Orthologous Genes

After the first discovery of the human *DDX11* gene by Frank and Werner [15], in the Lahti’s laboratory two distinct cDNAs were sequenced and found to be both related to yeast *CHL1* [1,2]. They were encoded by two highly similar genes (98% identity), named *CHLR1* (*DDX11*) and *CHLR2* (*DDX12*). The two genes were located in two adjacent regions of the human chromosome 12 (*12p11* and *12p13*, respectively) and proposed to be generated by a late event of gene duplication. Since sequences sharing high similarity with the 3′-terminal part of human *DDX11* were identified in the sub-telomeric region of several human chromosomes, it was postulated that the chromosome 12 locus containing *DDX11* and *DDX12* was subjected to many duplication events that were followed by translocation of the duplicated portions [16].

While, as previously mentioned, the human ChlR1/DDX11 protein was produced in recombinant form, purified and demonstrated to possess ATPase and DNA helicase activity in vitro, no report was published describing the production of the human ChlR2/DDX12 protein in recombinant form and it was not clear if the *ChlR2*/*DDX12* gene is truly expressed in mammalian cells or is an inactive pseudogene, as annotated in the databanks. Interestingly, the issue of the functionality of *ChlR2*/*DDX12* has been recently addressed in a work where it was demonstrated that a *DDX12* mRNA (encompassing a start codon, all exons and a poly-A tail) was transcribed in RPE1 cells, but a 5-bp deletion in exon 8 would lead to a predicted DDX12 protein that includes only a 300-residue N-terminal fragment of the DDX12 polypeptide chain and was expected to be devoid of any catalytic function [17]. Besides, these authors found that only ablating specifically *DDX11*, but not *DDX12*, with the CRISPR methodology in RPE1 cells proliferation defects and sister chromatid cohesion anomalies were observed, strongly suggesting that *DDX12* is not a functional gene.

## 4. Biochemical Features of the Human DDX11 DNA Helicase

To understand the physiological function of an enzyme, analyzing its substrate specificity is of fundamental importance. This is especially true in the case of nucleic acid helicases considering that the human genome encodes at least 95 putative helicases, among which 64 are predicted to be specific for RNA and 31 for DNA molecules. These enzymes are expected to act redundantly and/or selectively on DNA/RNA substrates having different structure/conformation.

After an initial biochemical characterization carried out in the laboratories of Lahti [2] and Hurwitz [5], which revealed for the first time that human DDX11 is an ATP-dependent DNA helicase able to translocate on single-stranded DNA with a 3′ to 5′ directionality, reaction requirements and substrate specificity of this enzyme were more extensively analyzed by Brosh and colleagues [6,7]. DDX11 was found to be able to unwind DNA molecules containing a single-stranded 5′-tail (with an optimal length of at least 15 nucleotides) required for helicase loading; in contrast, DNA duplexes having blunt ends or only a 3′-overhang are not melted [6]. Besides, DDX11 was found to resolve a three-stranded D-loop with an invading 3′-end but was not active on Holliday junctions. This substrate preference would suggest a role in homologous recombination (HR) reactions or telomere metabolism, due to the structural similarity between D-loops and T-loops present at the chromosomal ends.

Brosh and colleagues also examined the ability of DDX11 and other DNA helicases to unwind DNA duplexes containing damaged nucleotides such as 8,5′ cyclopurine deoxynucleoside (cPu) adducts, which are the products of oxidative damage and cause a modification of the DNA helix twist and base pairing stacking that can affect DNA replication and transcription. This study revealed that DDX11 was able to bypass and unwind DNA substrates containing cPu adducts on the translocating strand, while the DNA helicase activity of FANCJ and RECQ1 was completely inhibited by these oxidative lesions [18]. The same laboratory analyzed also the ability of various DNA helicases to unwind DNA substrates containing alkyl phosphotriester (PTE) lesions, which are produced by chemical genotoxic agents. It was found that the PTE lesions had an inhibitory effect only if they were located on the strand of the duplex DNA substrate, on which the DNA helicase translocates, whereas their presence in the displaced strand did not inhibit the unwinding activity of DDX11 and other SF2 DNA helicases. In contrast, the replicative hexameric DNA helicases of the archaeon *Methanobacterium thermoautotrophicum* (mini-chromosome maintenance-like, MCM, complex) and the eubacterium *Escherichia coli* (DnaB complex) were both found to be not inhibited by PTE adducts [19].

An interesting property of DDX11 and other SF2 Fe-S DNA helicases is the ability to displace proteins bound to DNA in an ATP-fueled reaction. This activity was analyzed by in vitro enzymatic assays carried out with synthetic biotinylated oligonucleotides bound to streptavidin. The physiological significance of the ability to actively displace DNA-bound proteins is not yet clear. However, this capability seems to be peculiar of the SF2 Fe-S cluster DNA helicases DDX11 and FANCJ, while some human RECQ-like DNA helicases do not display this enzymatic function [6,20].

A critical property of the DNA helicases that play a role in counteracting replication stress consists in their ability to untangle alternative DNA structures that can arise at genomic loci containing repetitive sequences (such as centromeres, telomeres, ribosomal (r)RNA gene clusters and fragile sites). These unconventional structures mainly consist of DNA containing triple-stranded (triplex) or G-quadruplex (G4) structures.

DNA triplexes, also named hinge DNA or h-DNA, are formed at poly(purine/pyrimidine)-rich regions in the human genome [21]. These alternative DNA structures, characterized by non-canonical Hoogsteen hydrogen bonding, can be formed intra- or intermolecularly. Intramolecular DNA triplexes form when an appropriate sequence partially melts with one of the two single strands folding back to complex with an adjacent duplex [22]. The physiological relevance of DNA triplexes was highlighted by immunofluorescence experiments with antibodies specific for these nucleic acid structures that revealed their presence in cells [23,24]. In vitro assays indicated that DDX11 is able to resolve inter- and intramolecular DNA triplexes with a catalytic efficiency much higher than the one displayed by other human DNA helicases (such as Werner, Bloom, DHX9 and FANCJ) [25,26,27]. The DDX11 helicase activity on DNA triplexes is ATP-dependent, has a 5′ to 3′ directionality and requires a 5′ single-stranded overhang on the third strand [25].

G4 structures can form at G-rich sequences due to the ability of guanine bases to form planar tetrads via Hoogsteen interactions [28]. This G-quartets are stabilized by metal ions (especially K^+^) and can stack through π–π interactions giving rise to G-quadruplex structures. G4 may have different strand composition: tetramolecular G4s contain guanine bases belonging to four parallel DNA strands; bimolecular G4s derives from two G-rich strands of DNA and unimolecular G4s are formed by folding on itself of a unique strand. Unimolecular G4 DNA structures are also characterized by length and sequence of loops that connect the G-quartets; besides, the DNA backbone can have different arrangements (parallel or antiparallel or mixed). While formation and stability of these peculiar DNA structures has been analyzed in vitro by many different sophisticated biophysical techniques, their physiological relevance has been a matter of debate. Only very recently a robust evidence in support of their existence and dynamic formation in living cells has derived from innovative single-molecule real-time imaging technique based on G4-specific fluorescent probe [29,30]. Several studies revealed that G4 structures might impact many aspects of the genome metabolism, including gene transcription regulation, replication origin definition and activation, DNA replication and telomere maintenance [31]. It is well established that occurrence of G-quartet stacks on DNA templates gives rise to replication stress because replicative DNA synthesis is inhibited by these alternate structures and the Cdc45/MCM2-7/GINS, CMG, complex, the eukaryotic, replicative DNA helicase is unable to resolve them when they are formed on the leading strand in front of the advancing replication machinery. The intervention of auxiliary DNA helicases, able to untangle these structures, is needed to remove these obstacles and alleviate the consequent replication stress. Human cells contain a plethora of DNA helicases that are able to dismantle DNA G4 structures in vitro with different specificity, including RECQ-like, SF2 Fe-S containing DNA helicases, DNA2 endonuclease/helicase, DHX36 and PIF1 [32,33,34,35,36].

**Figure 2:**
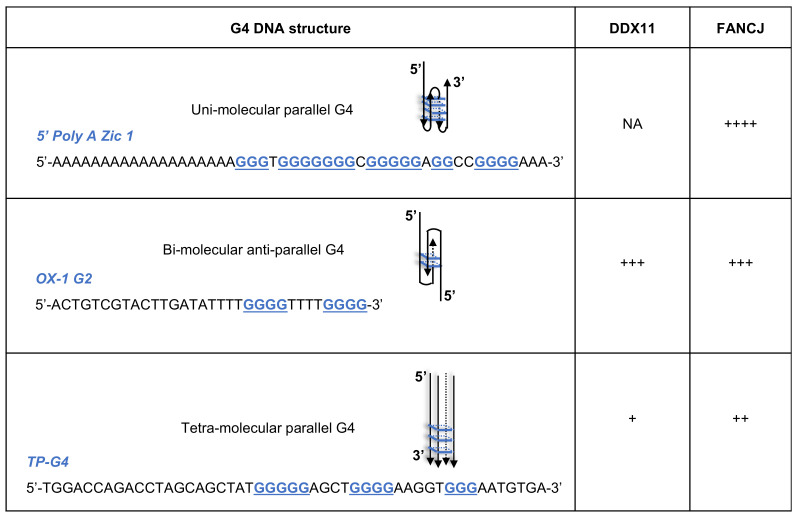
Specificity of DDX11 and FANCJ DNA helicases towards a set of G4-containing DNA substrates, whose structure is schematically depicted. The sequence of the corresponding synthetic oligonucleotides is also reported. Guanine residues forming the stacked G-quartets (colored in *blue* in the drawings) are highlighted in bold and in blue and underlined. Different catalytic efficiency of DDX11 and FANCJ in resolving the G4 DNA substrates is roughly indicated by a variable number of *+*, according to previous reports [6,7]. NA: not active.

Enzymatic assays carried out in vitro with G4 structures, which were prepared using synthetic oligonucleotides in the presence of K^+^ ions, revealed that DDX11 was able to efficiently dismantle only an alternative form of G–G-paired DNA, designated G2′ [6]. As schematically represented in Figure 2, this unusual G4 structure can occur when hairpin dimers of two antiparallel strands form Hoogsteen hydrogen bonds between guanine residues. The G2′ structure, predicted to arise from double T_4_-G_4_ repeats present in the *Oxytricha* telomeric sequence, was well characterized by both NMR studies [37] and X-ray crystallography [38,39]. In contrast, DDX11 was found to be almost completely inactive on parallel tetra-molecular G4s and unable to resolve parallel unimolecular G-quartet stacks formed by the Poly(A) Zic1-G4 DNA synthetic oligonucleotide derived from the sequence of the human zinc-finger protein of the cerebellum 1 (*Zic1*) 5′-end [40,41]. Both the human FANCJ [7] and DHX36 [41] DNA helicases were reported to resolve all these G4 structures in vitro with a high catalytic turn-over, pointing towards a key role of these enzymes in counteracting the stress caused by formation of these unconventional DNA structures at the replication forks.

## 5. Functions of DDX11 in DNA Repair and Replication Fork Protection against Replication Stress

The participation of DDX11 in DNA repair pathways based on HR in mammalian cells was revealed by the finding that loss of this protein caused a remarkable reduction of sister chromatid exchange events in HeLa cells treated with a chemical mutagen [42]. Besides, DDX11-knockeddown HeLa cells were highly sensitive to treatment with cisplatinum, a DNA cross-linking agent, and bleomycin, a radio-mimetic drug that induces DNA double-stranded breaks [42]. A study carried out by the Branzei group revealed that in DT40 cells DDX11 promoted repair of DNA bulky lesions (as the ones induced by methylmethane sulphonate, MMS) by HR and co-operated with the 9-1-1 checkpoint clamp and its loader, Rad17, to facilitate trans-lesion synthesis through abasic sites [43]. Besides, chicken DDX11 was found to operate in a pathway involved in repairing DNA interstrand cross-links that is parallel and redundant to the Fanconi anemia (FA) one.

As anticipated in the previous section, DDX11 plays a key role in counteracting replication stress due to the formation of unconventional DNA structures (such as triplexes and G4s) that block the smooth progression of the replisomes in the S phase. It was found that DDX11-depleted HeLa cells are highly sensitive to chemical agents (such as benzoquinoquinoxaline, BQQ) that bind and stabilize DNA triplex structures. Treatment of these cell lines with BQQ determined a remarkable increase of DNA triplexes, which were detected by indirect immunofluorescence with an antibody that specifically binds these structures [25]. In contrast, FANCJ-depleted cells were not sensitive to the action of BQQ and did not display an increased level of triplex DNA structures in their nuclei. These findings indicate that DDX11 may have a preponderant role in protecting our genome against the formation of triple-stranded DNA structures compared to FANCJ.

The Brosh group analyzed the sensitivity of various helicase deficient U2OS human cell lines to Telomestatin, a compound that specifically targets telomeric G4 structures [7]. In this study, DNA damage caused by Telomestatin was detected and quantitated by visualizing γ-H2AX foci by immunofluorescence. Cell lines, where DDX11 was depleted (along with *XPD*^−/−^ cells derived from a *Xeroderma pigmentosum* patient), were found to be resistant to a treatment with Telomestatin and did not display an increased DNA damage compared to control cells. In contrast, cell lines, where *FANCJ* was downregulated, displayed an increased level of γ-H2AX foci upon Telomestatin treatment compared to control cells. In parallel experiments, either DDX11- or FANCJ-depleted U2OS cells showed high sensitivity to mitomycin C (MMC), a DNA cross-linking agent [44].

A collaborative study carried out by Pisani and Brosh laboratories in 2016 revealed for the first time that DDX11 physically and functionally interact with Timeless, a component of the replication fork–protection complex [45]. DDX11 and Timeless were found to operate in the same pathway that preserves fork integrity in stressful conditions. These authors reported that the DNA helicase activity of DDX11 was remarkably increased in the presence of purified Timeless in assays carried out in vitro with various DNA substrates (forked duplex, three-stranded D-loop and G4-containing DNA) [45]. More recently Pisani and colleagues reported the identification of a DDX11 peptide responsible for the direct interaction with Timeless [9]. Its sequence is highly conserved in multiple alignments of the DDX11/Chl1 orthologs from yeast to humans and contains a short stretch of residues (the so-called “E-Y-E” motif) that is invariant in metazoan DDX11 orthologs. Mutagenesis of the “E-Y-E” motif strongly reduced direct interaction of DDX11 with Timeless either in vitro or in cell extracts [9], without affecting the protein DNA unwinding activity. Direct association to Timeless was reported to be essential for stable recruitment of DDX11 to the ongoing replication forks. Besides, the DDX11/Timeless interaction was also critical for sister chromatid cohesion and stable binding of the cohesin complex onto chromatin in S phase cells. In line with these findings, Sale and Pellegrini laboratories have recently reported that in DT40 cells the direct interaction between DDX11 and Timeless is critical for epigenetic mark inheritance at the *BU-1* chromosomal locus. This latter harbors a stable G4 structure located in front of the *BU-1* gene promoter that was proposed to be resolved by the combined action of chicken DDX11 and Timeless during passage of the replication fork [46]. Interestingly, in this study the authors also reported the identification and structural analysis of a novel Timeless C-terminal α-helical domain that could bind various kinds of G4 structures with high affinity. Thus, they proposed that maintenance of epigenetic marks at the *BU-1* site in DT40 cells requires a smooth progression of the DNA replication machinery at this genomic locus to allow an efficient recognition and resolution of the above G4 structure by DDX11 and Timeless. The results of this study are in line with a report showing that the ability of DDX11 to untangle G4-containing DNA substrates was stimulated in vitro in the presence of Timeless [45]. It is believed that another important player in this pathway could be the poly-ADP ribose polymerase 1 (PARP-1), which was found to be stably associated to Timeless in mammalian cells at laser-damaged chromatin sites [47,48]. Very recently, it was reported that G4 DNA structures bound and activated PARP-1 catalytic activity in vitro [49]. Understanding how DDX11, Timeless (together with the other components of the fork–protection complex), PARP-1 and the replicative DNA helicase (the CMG complex) cooperate at a stalled replisome to remove the G4 roadblock represents a very fascinating experimental challenge. Of note, in the DT40 cell system, co-depletion of both DDX11 and FANCJ had an additive effect on the loss of the epigenetic marks at the *BU-1* site indicating that these two DNA helicases might operate in independent parallel pathways important for G4 resolution at the replication fork [46]. These findings are consistent with a very recent study by the Job de Lange and Rob Wolthuis laboratory, where the effects of G4 stabilization on proliferation, chromosomal anomalies and DNA damage were analyzed in various DDX11-depleted cell lines (including lines derived from WABS patients) [17]. It was reported that the G4-stabilizers Quarfloxin and CX-5461 were both very toxic to cells lacking DDX11, but hardly affected *FANCJ*-knockout cells. In contrast, Telomestatin was found to be more harmful to FANCJ- compared to DDX11-deficient cells. Loss of both DDX11 and FANCJ had an additive effect on cell proliferation and accumulation of DNA damage after treatment with either Pyridostatin, another G4-binder, or MMC, suggesting again that DDX11 and FANCJ had redundant roles in counteracting the harmful effects of these drugs [17]. To explain the observed differences, it was pointed out that these compounds might target different G4 subsets depending on their structure/conformation and/or subcellular localization. In fact, Quarfloxin accumulates in the nucleolus, where it inhibits rRNA gene transcription by RNA polymerase I [50]; on other hand, Telomestatin targets the telomeric ends, where it inhibits the activity of telomerase [51]. Of note, DDX11, which was found in the nucleolus of interphase cells, was reported to be able to resolve only bimolecular antiparallel G4s with high catalytic efficiency, as previously described. While the G4 structures found at telomeres are unimolecular, antiparallel bimolecular G-quartet stacks are expected to be more abundant in the nucleolus, where the rRNA gene clusters are located [52].

## 6. Role of DDX11 in Chromatin Organization and Gene Transcription Regulation

DDX11 was proposed to have a role in the global organization of chromosomal territories and, consequently, regulation of gene accessibility and expression [53]. In fact, it was observed that in DDX11-depleted HeLa cells chromatin was decondensed in the perinuclear and perinucleolar regions, where it is normally localized. DDX11 was proposed to regulate the association of the heterochromatin protein-1 (isoform α, HP1α) at pericentric and telomeric sites of chromosomes. Mammalian HP1 proteins (isoforms α, β and γ) are involved in the assembly of higher-order chromatin structure and epigenetic inheritance. In particular, HP1α is responsible for recruiting SUV39H1 and SUV39H2 (the methyltransferases of histone H3) and DNMT1 and DNMT3a (the methyltransferases of the CpG DNA sequences) at specific genomic loci. At the same time, HP1α is able to specifically bind Lys9-methylated histone H3 (H3K9-me3) by means of its chromo-domain. These findings open up the possibility that DDX11 plays a role in regulating chromatin status and gene transcription by modulating recruitment of specific epigenetic modifiers [53].

As previously described (see Section 2), DDX11 was found to be present in nucleoli in interphase human cells. Besides, DDX11 was reported to promote transcription of rRNA genes and recruitment of UBF (upstream binding factor) and RPA194 (a subunit of the RNA polymerase I complex) at the 47S rRNA genes. Furthermore, loss of DDX11 caused a reduced phosphorylation and acetylation of UBF (modifications needed for activation of rRNA gene transcription); a reduced trimethylation of histone H3 Lys4 (H3K4me3) and an increased trimethylation of histone 3 Lys9 (H3K9me3). In DDX11-depleted cells alteration of these epigenetic marks caused a shift of the chromatin region that harbors the rRNA gene arrays from a euchromatic to a heterochromatic status leading to a defect in ribosome biogenesis [12]. In line with these findings, one of the hypotheses formulated about WABS etiopathogenesis is that this hereditary disease could derive from developmental defects due to a dysfunction in ribosome biogenesis, as described in Section 8.

## 7. Role of DDX11 in Sister Chromatid Cohesion

Sister chromatid cohesion is the process that ensures stable pairing of newly duplicated DNA molecules in each chromosome. In the eukaryotic cells the chromatid cohesion process is mediated by cohesin, a complex that contains a pair of rod-shaped SMC proteins (Smc1 and Smc3), whose association creates V-shaped heterodimers with ATPase domains at their ends connected by a kleisin subunit (Scc1) to form trimeric rings [54]. To these trimers additional hook-shaped proteins containing HEAT-repeats (named HAWKs, HEAT repeat proteins associated with kleisins) can be bound. Cohesin HAWKS are: Scc3, which is permanently associated, Pds5 and Scc2/NIPBL, which are interchangeable. Many additional cohesin regulators are also required to regulate the multiple cohesin functions during the different phases of the cell cycle [55]. In this context it is worth mentioning the acetyltransferase Eco1 of yeast cells (metazoan orthologs are Esco1 and Esco2), which is responsible for the site-specific acetylation of two critical Lys residues of the Smc3 subunit during cohesion establishment. As a consequence of this modification, the cohesin ring becomes resistant to the action of Wapl, an unloader factor. This latter counteracts the activity of the Scc2/NIPBL-Scc4 complex that is able to promote cohesin topological interaction with DNA by opening its ring-like structure at the Smc1–Scc1 interface. Smc3 acetylation and cohesion establishment are believed to take place at the replication forks. In fact, chromatid duplication and cohesion are concomitant events whose coordination is needed to ensure a faithful transmission of the genetic information during cell proliferation. A direct consequence of the functional coupling between DNA replication and cohesion is that downregulation of many replication factors (namely, Ctf4/AND-1, Chl1/DDX11, Csm3/Tipin, Tof1/Timeless, Mrc1/Claspin and Ctf18-RFC) gives rise to cohesion defects either in yeast or in mammalian cells [9,56].

As a matter of fact, the *CHL1* gene was originally identified in a work based on genetic screens in *Saccharomyces cerevisiae* of mutants that were unable to stably maintain genetic elements harboring autonomously replicating sequences (*ARS*) and displayed chromosome segregation defects [57,58]. Subsequent genetic analyses in yeast led to the identification of an inter-related synthetic lethal network among a number of replication factors revealing the existence of two epistasis groups: one including Ctf4, Chl1, Csm3 and Tof1 and the second containing Mrc1 and Ctf18-RFC [59].

As other studies in *S. cerevisiae* revealed an epistatic relationship also between *CHL1* and *FEN1*, which encodes the Flap endonuclease responsible for Okazaki fragment maturation [60], and in human cells DDX11 was demonstrated to bind Fen1 and enhance its catalytic activity in vitro [5], it was hypothesized that Chl1/DDX11 may play a role in cohesion establishment by executing a function at the replication fork lagging strand.

In a subsequent work carried out by the Uhlmann laboratory it was demonstrated that Chl1 associates to the ongoing replisomes by binding the homo-trimeric protein Ctf4 through a conserved Ctf4-interacting protein (CIP) box [61]. Ctf4 is considered to be a sort of landing pad for many replication factors, due to its ability to bind at least three different client proteins at the same time [62]. In the same work carried out by the Uhlmann group, it was found that the Chl1 helicase activity is not required for cohesion establishment, since a *CHL1* ATPase-dead mutant allele was able to correct the sister chromatid cohesion defects observed in *CHL1*^−/−^ cells [61]. Based on these findings, it was proposed that Chl1, in association with Ctf4, would anchor the cohesin rings at the replication fork stabilizing them in a conformation that would be more prone to being acetylated by Eco1. A subsequent elegant biochemical analysis carried in the same laboratory revealed that cohesin, once topologically bound to a duplex DNA, was able to entrap a second DNA molecule in a single-stranded form; subsequent conversion of this latter to a duplex molecule was needed to establish a stable DNA–DNA pairing. This cohesin-mediated process, named second-DNA capture, is ATPase-dependent, promoted by the Scc2–Scc4 loader complex and counteracted by the single-stranded DNA binding protein RPA [63]. Cohesion defects due to *CHL1* loss were not compensated in yeast cells that expressed an RPA mutant with lower single-stranded DNA binding activity *RPA1*^G77E^ suggesting that Chl1 and RPA acted in the same cohesion establishment pathway. It was postulated that Chl1 could bind cohesin at the replication fork and position it in a way favorable to entrap single-stranded DNA present on the lagging strand [63].

In a very recent elegant biochemical study carried out by the Nasmyth laboratory, it was demonstrated that in *S. cerevisiae* cells Chl1, together with the replication factors Ctf4 and Csm3/Tof1, belong to an epistasis group important for converting cohesin associated to the unreplicated DNA into a cohesive structure in a Scc2-independent process. Conversely, de novo cohesin loading at the replication fork required the interplay of Mrc1, the Ctf18-RFC complex and the Scc2 loader that defined a parallel independent cohesion establishment pathway. However, the molecular details of these processes have not yet been elucidated [64].

The role of the Chl1/DDX11 DNA helicase activity during the sister chromatid cohesion establishment process is controversial because, although DDX11 was found to be critical for cohesin association to chromatin in either yeast [65] or human cells [9], its ability to unwind duplex DNA was found to be not essential for chromatid pairing in *S. cerevisiae* [61]. Conversely, the catalytic activities of DDX11 were found to be critical for cohesion establishment in the avian cell system, as reported in a study by the Branzei group [43]. Although a not yet clarified helicase-independent function of Chl1/DDX11 in loading and/or retaining cohesin on chromatin cannot be completely ruled out, a recent comprehensive analysis of the molecular basis of WABS, describing newly diagnosed patients and the related *DDX11* pathogenetic variants, has revealed that the ability of DDX11 to resolve a specific subset of G4 DNA structures, abundant at rRNA gene arrays, was essential to prevent cohesion loss, replication delay, DNA damage and growth inhibition in many cell lines [17]. In this paper the authors proposed that the ability of DDX11 to dismantle peculiar G4 structures might prevent breaks and create enough “room” on the lagging strand to allow an efficient DNA entrapment by the cohesin rings [17]. These data indicate that DDX11 roles in DNA replication/repair and sister chromatid cohesion establishment are functionally linked and not distinguishable. However, elucidating the molecular mechanisms by which Chl1/DDX11 promotes pairing of the newly duplicated DNA molecules together with other components of the replication machinery represents an important future challenge for the researchers working in this field.

## 8. WABS Clinical Features

Warsaw breakage syndrome (WABS) is a very rare autosomal recessive disease, due to biallelic mutations of the gene coding for the DDX11 DNA helicase [66,67]. The clinical spectrum of WABS is heterogeneous with some cardinal symptoms observed in all patients including: (1) severe pre- and post-natal growth retardation, (2) microcephaly, (3) sensorineural hearing loss, (4) cochlear anomalies, (5) facial dysmorphia and (6) sister chromatid cohesion defects. This latter clinical manifestation led to the notion that WABS is a cohesinopathy, even if not all the cohesinopathies are characterized by a precocious chromatid separation cellular phenotype [68]. Cohesinopathies are genetic diseases caused by mutations in genes involved in the sister chromatid cohesion process, including: Cornelia de Lange syndrome (CdLS), caused by mutations in genes encoding the cohesin structural components (*SMC1A*, *SMC3* and *RAD21*) and regulators (*NIPBL* and *HDAC8*); Roberts syndrome (RBS), due to mutations of the cohesin acetyl-transferase gene (*ESCO2*) and chronic atrial and intestinal dysrhythmia (CAID) syndrome, linked to mutations of the *SGOL1* gene encoding Shugoshin [68,69]. It should be pointed out that sister chromatid cohesion defects are observed in WABS and RBS patient cells, but not in those taken from CdLS or CAID probands. This can be due to the multiple functions played by the cohesin complex that are differentially affected in the various “cohesinopathies” [68,69,70]. The cohesion defects observed in metaphase chromosome spreads of WABS (and also RBS) immortalized fibroblasts mainly consist in a characteristic “railroad” configuration of the paired sisters with the centromere constriction that seems to be loosened (premature centromere division, PCD). This peculiar morphology is likely due to repulsion of the corresponding centromeric heterochromatic regions. Besides, in a not negligible percentage of WABS- and RBS-derived cells, all the sister chromatid pairs are completely disjointed, a phenotype known as premature chromatid separation (PCS). Interestingly, the percentage of WABS patient fibroblasts displaying these chromosomal anomalous morphologies is remarkably increased following treatment with various genotoxic agents (including MMC, Camptothecin, and the G4-binders, Pyridostatin, Quarfloxin and CX-5461) [8,17]. Similarities among WABS, RBS and CdLS include various degrees of cognitive impairment and facial anomalies. However, WABS patients do not display limb reduction as usually observed in RBS and CdLS probands, although finger abnormalities are quite common to all these pathologies. Besides, an important diagnostic overlap is observed between WABS and FA. This latter is a recessive genetic disorder characterized by growth retardation, microcephaly, bone malformations, progressive bone marrow failure and an increased cancer predisposition. Due to this clinical complexity, FA diagnosis is based on the detection of increased chromosome breaks/gaps, upon administration to patient lymphocyte cultures of a DNA cross-linking agent, such as diepoxybutane (DEB) or MMC. FA patient cells show a very high sensitivity to these genotoxic agents, a feature that is also observed in cells derived from individuals suffering from other chromosomal instability disorders, including WABS, RBS and Nijmegen breakage syndrome (NBS). Therefore, the use of the chromosomal breakage test could lead to a misdiagnosis of FA, also due to other clinical manifestations common to all these genetic syndromes [71]. In particular, altered skin pigmentation and short stature were described in a high percentage of FA patients with mutation of the *FANCA* gene (complementation group A); whereas other skeletal defects (affecting the radius and/or thumb) were found in a small proportion of FA patients classified in different complementation groups. All these symptoms are also common to many WABS probands. Of note, it has been recently reported that another clinical feature is shared by WABS and FA patients: altered mitochondrial metabolism [14,72,73]. WABS cells displays a marked reduction of oxygen consumption and ATP synthesis in the presence of pyruvate/malate, a partial impairment of electron transport between complexes I and III, similarly to what observed in FA cells, and a remarkable decrease of complex IV activity, which is not displayed by FA cells [74,75]. However, the ultrastructure of mitochondria in WABS patient cells appeared to be normal with parallel *cristae* across the entire body and a dense mitochondrial matrix, in contrast to what observed in FA cell lines [14,72,73]. Therefore, due to the clinical overlap of WABS with other genome instability hereditary syndromes (especially FA), clinicians should analyze metaphase chromosome spreads for detecting possible cohesion defects, which are only observed in WABS (or RBS) patient cell lines, to avoid any possible mistake in diagnosis [71].

**Figure 3:**
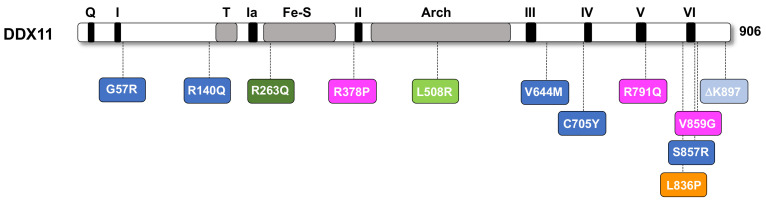
Amino acid substitutions of DDX11 found in Warsaw breakage syndrome (WABS) patients. A schematic representation of the human DDX11 polypeptide chain is reported. Color code is as in Figure 1 for black and grey elements. DDX11 pathogenic mutations described in the same article are highlighted with the same color: light blue [8]; indigo [17]; dark green [76]; light green [77]; pink [78]; orange [14].

To date, 23 cases of WABS were described in the medical literature starting from the first diagnosed individual, a boy from Warsaw (Poland) that was studied by the de Winter group in Amsterdam and inspired the disease name [8]. The amino acid changes produced by the different pathogenic *DDX11* missense mutations are schematically reported in Figure 3. Most of the mutated amino acid residues map within the conserved helicase motifs and the relevant mutations are expected to impair the catalytic functions of the DDX11 DNA helicase. For a certain number of *DDX11* pathogenic missense alleles this was tested by in vitro enzymatic studies of the mutant proteins, which were produced in recombinant form and purified.

In particular, the following amino acid substitutions were found to compromise the ability of DDX11 to unwind forked duplex DNA substrates: ∆K897 [8], R263Q [76], L836P [14] and C705Y [17]. Of note, the DDX11 mutation R140Q, which was described in a recently discovered WABS affected individual (WABS03 patient) can be considered as a variant of unknown significance (VOUS), as it does not map in any conserved sequence motif of the DDX11 polypeptide chain and, moreover, the corresponding mutated protein displayed a level of helicase activity comparable with the wild type counterpart in assays carried out in vitro with a flayed duplex DNA substrate; in addition, its cellular localization and stability, measured in cells treated with proteasome inhibitors, were found not to be altered compared to wild type DDX11 [17]. Other WABS pathogenic missense mutations are R378P, R791Q and V859G [78]. The catalytic activities of the corresponding DDX11 mutant proteins were not studied, but the amino acid residues R791 and V859 are both located in close proximity of conserved helicase box V and VI, respectively, and are, thus, expected to reduce DDX11 DNA unwinding activity (see Figure 3). Conversely, the DDX11 R378P mutant, whose amino acid change does not involve any conserved helicase motif, was found to cause a severe protein destabilization. It was proposed that the DDX11 R378P mutant underwent to an extensive proteolytic degradation, likely due to misfolding of its polypeptide chain, as revealed by finding that the protein was stabilized in cells that were treated with MG132, a ubiquitin-proteasome inhibitor [78]. It is interesting to notice that in all the WABS patient derived cell lines that were analyzed by immunoblot experiments the expression level of the endogenous DDX11 was found to be remarkably reduced, suggesting that the pathogenic *DDX11* missense alleles are hypomorphic, since they encode unstable and/or inactive (or partially active) proteins (with the exception of the DDX11 R140Q mutant that seems to behave like the wild type protein, as mentioned above).

## 9. WABS Etiopathogenesis and Studies of Animal Disease Model Systems

**Figure 4:**
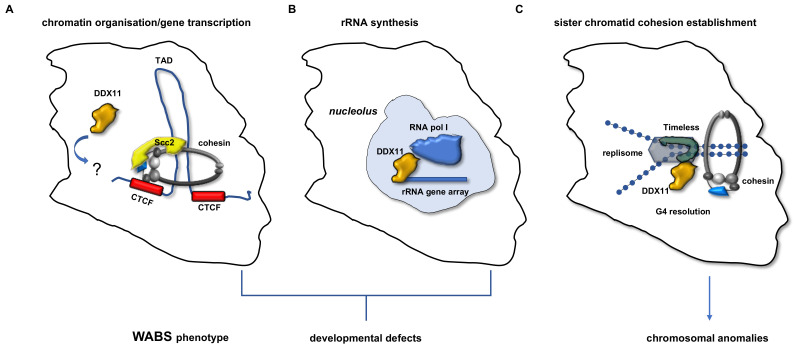
DDX11 cellular roles and related phenotypes observed in WABS patients. (**A**) Formation of chromatin TADs (topologically associating domains) in interphase cells is due to the loop extrusion activity of the cohesin-Scc2 complex. TAD boundaries are defined by the CCCTC-binding factor (CTCF), a sequence specific DNA binding protein that acts as a chromatin insulator [84]. A possible function of DDX11 in promoting cohesin loop extrusion activity has not yet been demonstrated. Nevertheless, a role of DDX11 in chromatin spatial organization at pericentromeric and sub-telomeric regions was proposed [53]. The DNA binding mode of the cohesin-Scc2 complex is only indicative. (**B**) DDX11 was proposed to recruit the RNA polymerase I complex at the rRNA gene arrays in the nucleolus and to promote rRNA synthesis [12]. DDX11 loss produces shortage of the ribosome pool and a general reduction of the mRNA translation causing the birth defects observed in WABS probands. (**C**) DDX11 promotes sister chromatid cohesion establishment with the help of Timeless during S phase [9]. Resolution of G-quadruplex structures (especially at the rRNA gene arrays and, possibly, at other difficult-to-replicate templates) assists DNA entrapment by cohesin by a not yet clarified mechanism (see text for details).

Attempts to create a mouse WABS model system were unsuccessful because knock-out of the *DDX11* gene led to lethality of murine embryos at day *E10.5* and placental malformations [79]. Besides, induction of random mutations in mice by administration of N-ethyl-N-nitrosourea (ENU) produced a *DDX11* missense mutation leading to an amino acid change (L743P) within the conserved helicase motif V that also was not compatible with embryo development after day *E8.5* [80]. More recently, mice harboring a *DDX11* G57R mutant allele, corresponding to the one found in a WABS patient and predicted to encode an ATPase-dead protein, were generated (*DDX11*^G57R/WT^), but, when they were intercrossed, the offspring with a *DDX11*^G57R/G57R^ genotype was unable to overcome day *E10* of the embryonic development, indicating that a functional DDX11 DNA helicase is essential for life [17]. More successful were the attempts to create a zebrafish model of WABS by downregulating *DDX11* mRNA translation with morpholino oligomers [12]. Loss of DDX11 in zebrafish embryos provoked a growth defect and various vertebral and craniofacial anomalies with shortened and twisted torsos, longer faces, smaller eyes, low and protuberant mouths and narrowed eye distances, all features resembling the ones described for some WABS patients. Besides, modifications of the epigenetic status at the rRNA gene arrays were found in DDX11-depleted zebrafish embryos, together with a reduced recruitment of RNA polymerase I to the promoter region of these genes and a consequent decreased production of rRNA precursors [12]. Based on these findings it was postulated that WABS could be due to a malfunctioning of the nucleolus: the consequent reduced production of functional ribosomes would hamper protein biosynthesis and cellular proliferation during early stages of the embryonic development. This led to the proposal of considering WABS and other cohesinopathies as ribosomopathies, a spectrum of rare diseases due to defects in ribosome biogenesis and function (see Figure 4) [81,82,83].

Ribosomopathies are caused by mutations in genes coding for ribosomal proteins or for protein factors involved in maturation or modification of rRNAs [85]. Ribosomes are key players in messenger RNAs (mRNAs) translation, an essential process that takes place in the cytoplasm of the eukaryotic cells. They are large ribonucleoprotein complexes consisting of four RNA molecules (25S, 18S, 5.8S and 5S rRNA) and about 80 proteins that assemble to form two subunits (named 40S and 60S or small and large ribosomal subunit, respectively). Ribosome biogenesis is an elaborated and energy consuming process that requires the coordinated function of more than 200 proteins and occurs both in the cytoplasm and in the nucleolus, where rRNAs, the most abundant fraction of RNA in the eukaryotic cell, are transcribed from the about 600 ribosomal DNA (rDNA) repeats. The nucleolus is a membrane-less nuclear compartment that contains the nucleolar organizing regions (NORs), where rDNAs arrays from different chromosome loci are clustered. In human cells, NORs are located in the short arms of the acrocentric chromosomes 13, 14, 15, 21 and 22, which harbor the genes encoding the 28S, 18S and 5.8S RNA, whereas the 5S rRNA gene repeats map exclusively on chromosome 1 [86]. Haploinsufficiency for the ribosomal protein genes underlies several ribosomopathies including Diamond Blackfan anemia, 5-q syndrome and Shwachman Bodian Diamond syndrome. All these pathologies are characterized by a variety of skeletal or craniofacial abnormalities and different forms of anemia and other hematological symptoms. Conversely, no bone marrow failure phenotypes but only cranofacial growth defects are observed in individuals affected by the Treacher Collins syndrome, a ribosomopathy caused by mutations of genes coding for subunits common to RNA polymerase I and III or for the ribosome biogenesis factor Treacle (*TCOF1*), a dense fibrillary component of the nucleolus [85]. An important observation is that ribosomopathies and cohesinopathies (including WABS) display common clinical manifestations encompassing prenatal and postnatal growth retardation, microcephaly and craniofacial abnormalities. Of note, as previously discussed, in a zebrafish model of WABS it was found that DDX11 loss gave rise to a decreased level of rRNAs due to a reduced recruitment of RNA polymerase I to the promoter region of the corresponding genes. Based on these findings, it was postulated that WABS could be due to an abnormal functioning of the nucleolus in *DDX11*-mutated cells. Besides, nucleolar anomalies are also observed in cells where subunits of the cohesin complex are mutated and chromosomal cohesion loss leads to a remarkable reduction of rRNA synthesis and defect in ribosome biogenesis in these cell lines [87,88]. Collectively, these data support the proposal that “cohesinopathies” and “ribosomopathies” share a common etiology, as they would derive from defective protein biosynthesis [81,82]. Nevertheless, how deregulation in ribosome biogenesis impairs cellular functions in these pathologies and why their symptomology displays such a high variability still remain elusive. One hypothesis (the “ribosome concentration model”) predicts that a decreased level of functional ribosomes leads to a global reduction of mRNA translation efficiency and, in turn, to the embryonic developmental defects that are common to all these hereditary diseases. Another proposal envisages that accumulation of unused ribosomal proteins, due to a dysregulated biosynthesis, prevents ubiquitination of p53 by E3 ubiquitin-protein ligase MDM2 and its subsequent degradation, and, in turn, promotes cell cycle arrest and apoptosis (“p53-mediated model”). This would mainly affect fast-dividing cells such as hematopoietic and embryonic cells, accounting for the bone marrow failure phenotypes, specifically observed in most ribosomopathies, and the developmental defects, found in either ribosomopathies or cohesinopathies [85].

An alternative theory about the molecular bases of WABS and other “cohesinopathies” is that these diseases could mainly derive from a dysregulated expression of genes that are critical for embryonic development [89]. Recent studies have revealed important functions of cohesin and the network of cohesin regulators/modifiers in organizing the chromatin loops (named topologically associating domains, TADs), which are believed to play a role in regulating gene transcription programs during development [84,90,91]. Global transcription alterations were described in CdLS and other “cohesinopathies” leading to the notion that they could be all disorders of transcriptional regulation or “transcriptomopathies” [92,93]. Consistently with this hypothesis, DDX11 was demonstrated to play a role in regulating the status of chromatin by recruiting the HP1α factor at pericentromeric and sub-telomeric regions in HeLa cells [53]. This finding suggests that DDX11 may be involved in chromosome architecture maintenance, as previously described (see Section 5). Besides, it was proposed that DDX11 could have a role in promoting stable binding of cohesin to chromatin in HeLa cells [9], in line with a previous report showing that in budding yeast association of the cohesin loader Scc2 is strikingly reduced in the absence of Chl1 during the S phase [94]. Nevertheless, a function of DDX11 in promoting the DNA loop extrusion activity of the human cohesin-Scc2 complex has not yet been discovered (see Figure 4). Instead, DDX11 has been found to be crucial in resolving alternative DNA secondary structures and assisting DNA entrapment by cohesin rings at the replication forks in many cell systems, as discussed in Section 6. However, it cannot be ruled out that WABS and other cohesinopathies might have a complex etiology and the developmental defects that characterize all these rare genetic syndromes could be due to either translational dysfunction (as in the ribosomopathies) or transcriptional dysregulation (as in the transcriptomopathies).

## 10. Conclusions and Epilogue

Herein, we aimed to review recent findings about the human genome stability maintenance DNA helicase DDX11 and the related rare genetic disease, Warsaw breakage syndrome, which is characterized by sister chromatid cohesion anomalies and developmental defects. WABS etiopathogenesis has not yet been elucidated, mainly due to a lack of disease animal models and limited knowledge of the molecular and cellular functions of DDX11. The recent finding that in human cells loading of cohesin onto chromatin may be promoted by DDX11 opens up the possibility that non-canonical functions of cohesin, in particular formation and stabilization of chromatin loops, could be regulated by DDX11 [9]. Creation of different WABS mouse models, where the *DDX11* gene is conditionally knocked-out, is expected to reveal a critical role for this DNA helicase in the deployment of evolutionarily conserved gene transcription programs during embryonic development. At the same time, single-molecule biophysical studies with purified proteins and protein complexes will allow us to elucidate a putative role of DDX11 in modulating the different DNA-binding modes of the cohesin complex. The results of these in vitro and in vivo complementary studies will provide insight into the molecular bases of WABS and other diseases that are due to mutation/loss of genes belonging to the chromosomal cohesion pathway.
